# Improved flooding tolerance and carbohydrate status of flood-tolerant plant *Arundinella anomala* at lower water temperature

**DOI:** 10.1371/journal.pone.0192608

**Published:** 2018-03-21

**Authors:** Xiao qi Ye, Jin liu Meng, Bo Zeng, Ming Wu

**Affiliations:** 1 Institute of Subtropical Forestry, Chinese Academy of Forestry/ Research Station of Hangzhou Bay Wetlands Ecosystem, National Forestry Bureau, Fuyang, China; 2 Key Laboratory of Eco-environments in Three Gorges Reservoir Region (Ministry of Education), Chongqing Key Laboratory of Plant Ecology and Resources in Three Gorges Reservoir Region, School of Life Sciences, Southwest University, Chongqing, China; Universidade do Minho, PORTUGAL

## Abstract

**Background:**

Operation of the Three Gorges Reservoir (TGR, China) imposes a new water fluctuation regime, including a prolonged winter submergence in contrast to the natural short summer flooding of the rivers. The contrasting water temperature regimes may remarkably affect the survival of submerged plants in the TGR. Plant survival in such prolonged flooding might depend on the carbohydrate status of the plants. Therefore, we investigated the effects of water temperature on survival and carbohydrate status in a flood-tolerant plant species and predicted that both survival and carbohydrate status would be improved by lower water temperatures.

**Methodology:**

A growth chamber experiment with controlled water temperature were performed with the flood-tolerant species *Arundinella anomala* from the TGR region. The plants were submerged (80 cm deep water above soil surface) with a constant water temperature at 30°C, 20°C or 10°C. The water temperature effects on survival, plant biomass and carbohydrate content (glucose, fructose and sucrose and starch) in the viable and dead tissues were investigated.

**Principal findings:**

The results showed that the survival percentage of *A*.*anomala* plants was greatly dependent on water temperature. The two-month submergence survival percentage was 100% at 10°C, 40% at 20°C and 0% at 30°C. Decreasing the water temperature led to both later leaf death and slower biomass loss. Temperature decrease also induced less reduction in glucose, fructose and sucrose in the roots and leaves (before decay, p < 0.05), but only marginally significant in the stems (p < 0.05). However, the starch content level did not differ significantly between the water temperature treatments (p > 0.05). Different water temperatures did not alter the carbon pool size in the stems, leaves and whole plants (p > 0.05), but a clear difference was found in the roots (p < 0.05), with a larger pool size at a lower temperature.

**Conclusions/Significance:**

We concluded that (1) *A*. *anomala* is characterized by high flooding tolerance and sustained capability to mobilize carbohydrate pool. (2) The survival percentage and carbohydrate status of submerged *A*. *anomala* plants were remarkably improved by lower water temperatures. The survival of submergence seemed to be closely associated with the sugar content and carbohydrate pool size of the roots, which contained the lowest amount of carbohydrates. Three Gorges reservoir impoundment in winter is beneficial to the survival of submerged *A*. *anomala* in riparian area of the reservoir due to the low water temperature.

## Introduction

The construction of the Three Gorges reservoir (Yangtze River, China) caused many environmental changes to local ecosystems. One of these changes was altered water fluctuation timing and water temperature (S1 Fig). Natural flooding in Yangtze River occurs in summer, with mean air temperature of approximately 25 °C from June to September; the water level can rise by 10 meters and lasts for 1–2 weeks at each flood event. After the construction of the reservoir, the water level of the Yangtze River in the reservoir is manipulated by reservoir regulation, which maintains at 175 m (above the sea level) for approximately 6 months in winter (mean air temperature lower than 15 °C) and 145 m (above the sea level) in summer. This dramatic change in water level fluctuation timing and water temperature may have strong impacts on the riparian vegetation of the reservoir. *Arundinella anomala*, a grass species from the riparian area of the reservoir, is tolerant to river flooding than other species. Previous study showed that *A*. *anomala* can survive complete submergence in summer for up to 60 days [1], but how this species survives prolonged winter submergence is unknown.

Flooding is a stress for most terrestrial plants. Two major problems for submerged terrestrial plants arise from the decreased gas exchange between the plants and their environment [2]. First of all, in terrestrial plants, carbon assimilation and respiration are usually reduced after flooding. Photosynthetic activity is decreased to various extents depending on the flooding regimes [1,3]. Waterlogging (in which only the belowground part of the plant is submerged) usually decreases photosynthetic capacity [4], and complete submergence of terrestrial plant species could arrest photosynthesis immediately due to low light and CO_2_ availability in the water, although limited underwater photosynthesis could possibly happen [5,6]. Secondly, oxygen deficiency due to submergence not only causes injuries to plant tissues but also decreases the energy yield efficiency from sugar catabolism, which increases the demand for sugar utilization [7]. Despite reduced metabolism intensity in flooded plants [8], cumulative carbohydrate loss due to respiration could lead to tissue starvation and death [7]. It is expected that carbohydrates may be critically important for the survival of flooded plants.

Previous studies have shown that flooding survival is highly dependent on the species as well as the flooding stress intensity, such as duration and water depth [9,10] and other factors. The survival of several grassland species in winter flooding in the River Rhine was better than in summer flooding [11]. Water temperature, one of the important factors affected by flood timing, determines the loss of biomass and respiration of carbohydrates [12]. Recent studies also showed that *Populus deltoids* and *Salix variegata*, two riparian tree species, can well survive winter flooding [13, 14]. These results suggest the importance of the temperature dependent effects on carbohydrate utilization to flooding tolerance. However, how and why the survival percentage is altered by different water temperatures needs to be further investigated.

In this study, *A*. *anomala* plants were exposed to prolonged flooding at three water temperatures. The survival percentage after long-term flooding and carbohydrate content fluctuation during the whole flooding period were measured. We propose that survival from prolonged flooding could be partly explained by effects on the carbohydrate status of flooded plants with death from submergence being accelerated by higher water temperatures. The results of this study may be useful for predicting and understanding the performance of *A*. *anomala* in the riparian area of the Three Gorges reservoir.

## Materials and methods

### Growth conditions and plants

Single ramets with similar shoot heights of 10–15 cm were transplanted into pots (9 × 9 ×10 cm) filled with a mixture of potting soil and sand (1:1, V: V). The transplanted ramets grew in a greenhouse with a day/night temperature of 19/17°C and an ambient day length for two months (December, 2007 to January, 2008). After that, the plants were transferred to an indoor laboratory for one month before submergence treatments started with an air temperature that was constant at 20°C and photosynthetically active radiation (PAR) at 102 μmol photons m^-2^ s^-1^.

### Treatments

The submergence treatments in the growth chamber used a total of 128 plants. At the beginning, eight plants were harvested for initial biomass and carbohydrate content analyses. The remaining plants were randomly divided into four groups with 30 plants each. One group of plants, which was regularly watered and grown in well-drained soil, was the control plant group. The other three groups of plants were completely submerged in six plastic containers (each group had 15 plants in 250 liter containers with a water depth of 80 cm) filled with tap water. Water in the containers was saturated with air by continuous and gently pumping of air bubbles through the water. Water temperature was regulated at either 30°C, 20°C or 10°C with two replicate containers per water temperature. For the 30°C treatments, the water temperature was maintained by two heating sticks. The water temperature was maintained at 10°C by circulating cooled water in plastic tubes in the containers. The pH of the tap water was 8.2–8.4 during the entire treatment period. The illumination just above the water surface was the same as the control plants.

Five plants were harvested on days 10, 30 and 60 from each of the four treatments. Plant tissues were collected separately for the leaf blades, leaf sheathes, stems and roots; and then, the tissue was freeze-dried and ground. The treatments were terminated on day 60. The remaining 15 plants from each treatment were taken out of the water and placed under the same growth conditions as the control plants. After two months recovery period, for determination of survival percentage, the number of living plants and dead plants were recorded. Plants which had not developed viable buds and green leaves were considered dead. The survival rate was calculated as the ratio of the number of living plants to the total number of plants in each treatment (15 plants).

### Sugars and starch measurements

We found only three major sugars in the *A*. *anomala* extracts via HPLC analysis: glucose, fructose and sucrose. Consequently, only those three sugars and starch were measured in this experiment. For each ground sample, approximately 30 mg of freeze-dried plant material was weighed, and the soluble sugars were fully extracted three times in distilled water for 30 min at 80°C. Glucose, fructose and sucrose in the extracts were analyzed with a coupled enzyme assay [15] using a multiplayer photometer (Anthos Mikrosysteme GmbH, Krefeld, Germany). For starch analysis, another two portions (each 30 mg) from each sample were weighed and autoclaved with distilled water for 2 h at 120°C, 1 bar. After this, one of these two autoclaved samples was incubated in Na-acetate buffer (50 mM, pH 4.9) with 1.4 U amyloglucosidase and 1 U α-amylase) at 37°C for 16 h, and the other autoclaved portion was incubated with the same volume of distilled water. The glucose content was then determined using the same procedure as described above for these two samples. The starch content was calculated as the difference in the total glucose content between the enzyme-incubated and water-incubated samples. The total carbohydrate pool was defined as the sum of glucose, fructose, sucrose and starch.

### Statistics

All the statistical analyses were performed in Sigmastat 2.03 (SPSS Inc., 1992–1997). The significance level was set at 0.05. The effects of the submergence treatments (control and submergence at water temperatures of 10°C, 20°C, 30°C) and the treatment duration (0, 10, 30, 60 days) on biomass, carbohydrate level and the carbohydrate pool were evaluated with a two-way ANOVA. Within one flooding period, or within one water depth, a multi-comparison was conducted.

## Results

**Fig 1:**
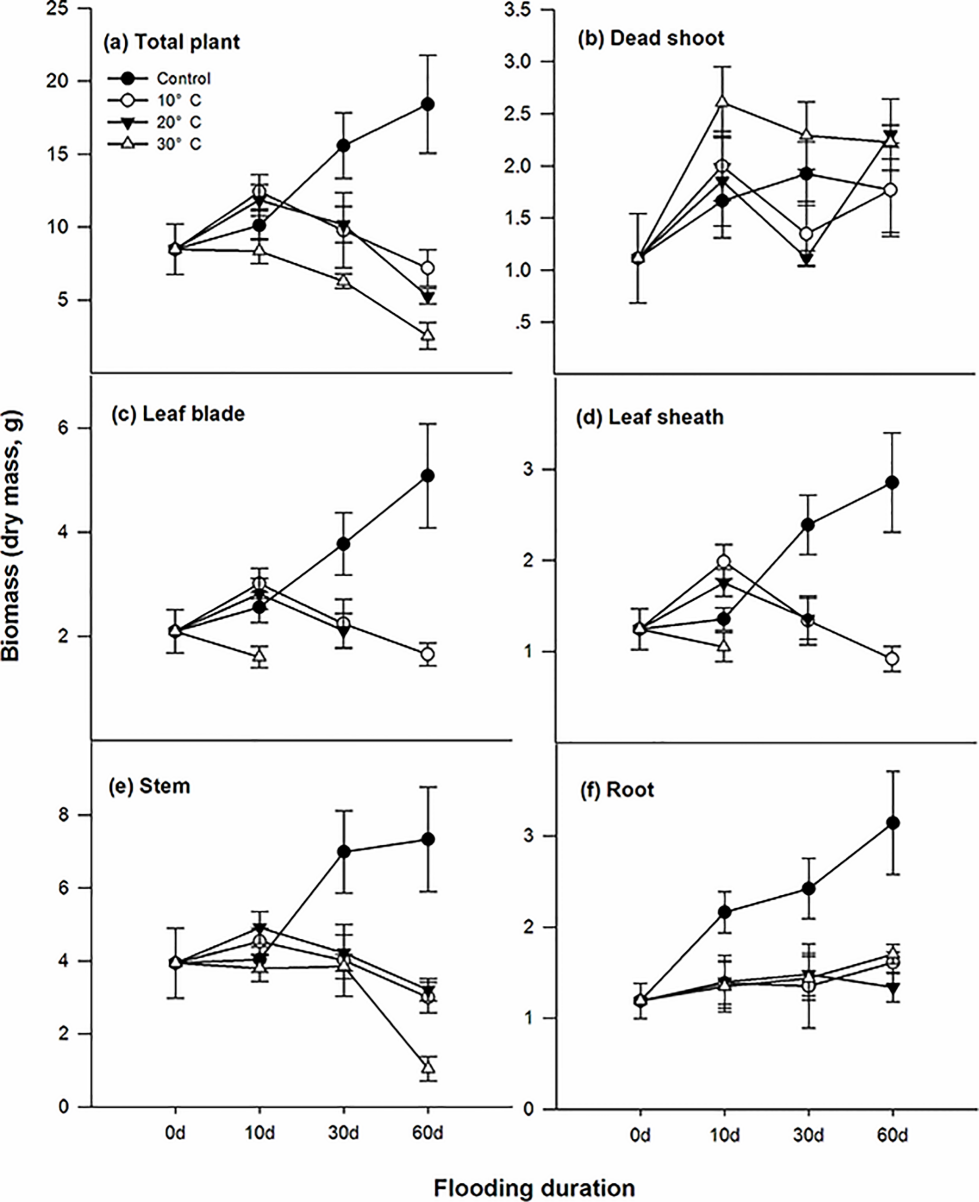
Biomass (dry mass DM, g) of the stems (a), roots (b), leaf blades (c), leaf sheathes (d), whole plant (e, excluding the dead shoot) and dead shoots (f) of *A*. *anomala* individual plants growing in either well-drained soil or submerged at water temperatures of 10°C, 20°C or 30°C in Experiment II. The missing data for the living biomass of the leaf blade and leaf sheath is due to leaf decay by submergence (*n* = 5, points = means ± SE).

The water temperature had a significant effect on the survival percentage after two months of submergence. With the increasing water temperature, the survival percentage decreased from 100% in the 10°C treated plants to approximately 40% at 20°C and 0% at 30°C. The total living biomass of the individual plants increased in the non-submergence condition and decreased in the submergence conditions, but the difference was only significant from day 0 at 30°C (p < 0.05, Fig 1a). This loss of biomass in the submerged plants was mainly due to the death of the shoots (Fig 1b). In the submerged plants at 30°C, all the leaves decayed before day 30 and the same symptoms were observed before day 60 at 20°C (Fig 1c and 1d). Plants submerged at 10°C maintained most of their leaves (Fig 1c and 1d). The stem biomass did not decrease significantly with the submergence treatments, except for a very low decrease at 30°C at day 60 (p < 0.05, Fig 1e). In contrast to the other tissues, the biomass accumulation in the roots was strongly and rapidly reduced by submergence, but root biomass did not decrease further and remained constant, independent of the water temperature (p > 0.05, Fig 1f).

**Fig 2:**
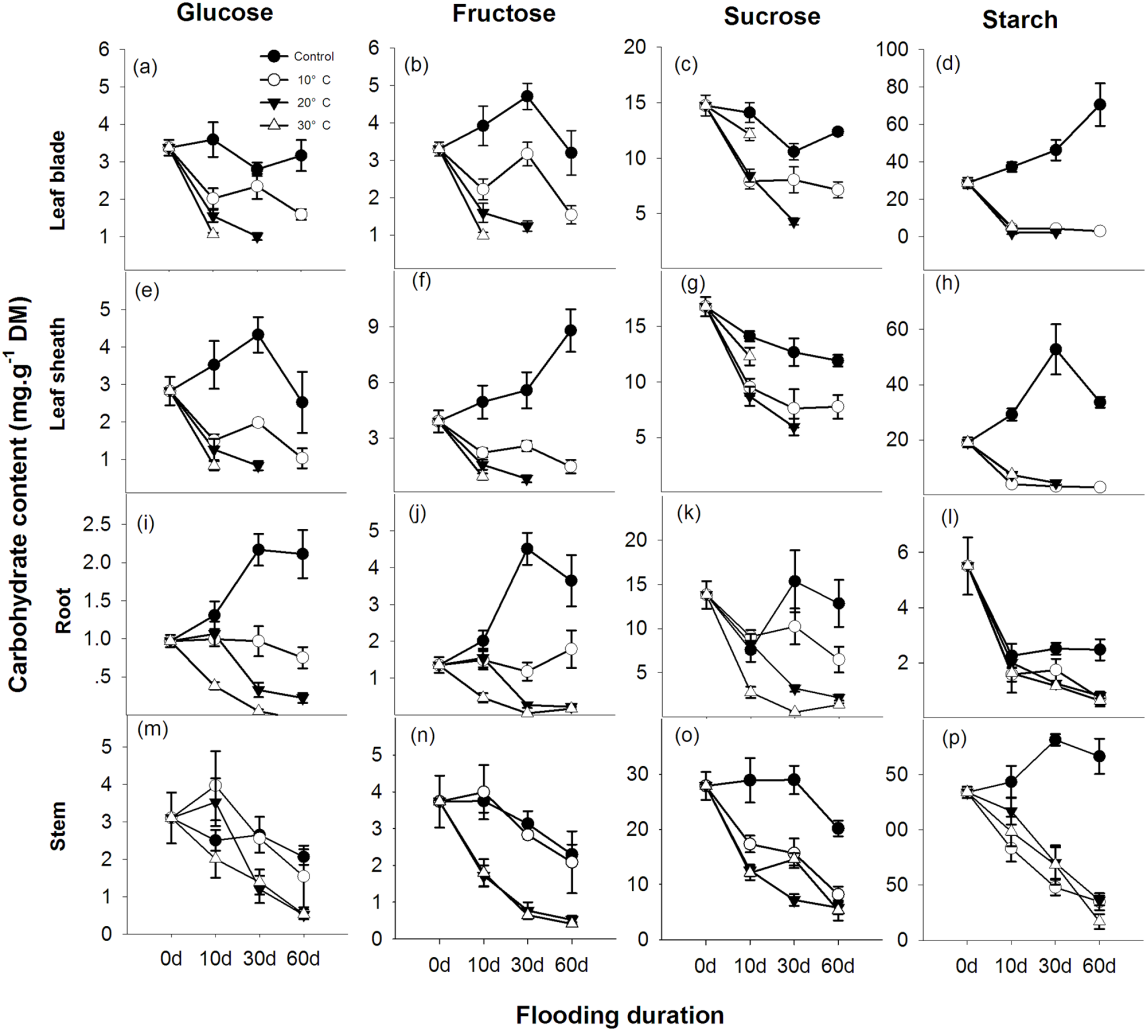
Glucose, fructose, sucrose and starch (mg·g^-1^ DM) content in the leaf blades (a–d), leaf sheathes (e–h), roots (i–l) and stems (m–p) of *A*. *anomala* plants growing in either well-drained soil (closed circle) or submerged at water temperatures of 10°C, 20°C or 30°C in Experiment II. The missing data for the carbohydrate content of the leaf blades and leaf sheathes occurred due to leaf decay from submergence (*n* = 5, points = means ± SE).

Submergence decreased the sugar and starch content in all tissues, independent of the water temperature (p < 0.05, Fig 2). The decreased level of carbohydrates was dependent on the submergence duration and water temperature (p < 0.05, Fig 2). In the leaves (Fig 2a–2h) and roots (Fig 2i–2l), the decrease was remarkably fast during only the first 10 days of submergence, but in the stems (Fig 2m–2p), the decrease of carbohydrates was fast and continuous during the entire treatment period. The temperature effects were most pronounced in the leaves and roots and were less pronounced in the stems. Generally, a lower water temperature led to higher sugar content. No temperature effect was found in the starch level for any tissue (p < 0.05, Fig 2d, 2h, 2l and 2p).

**Fig 3:**
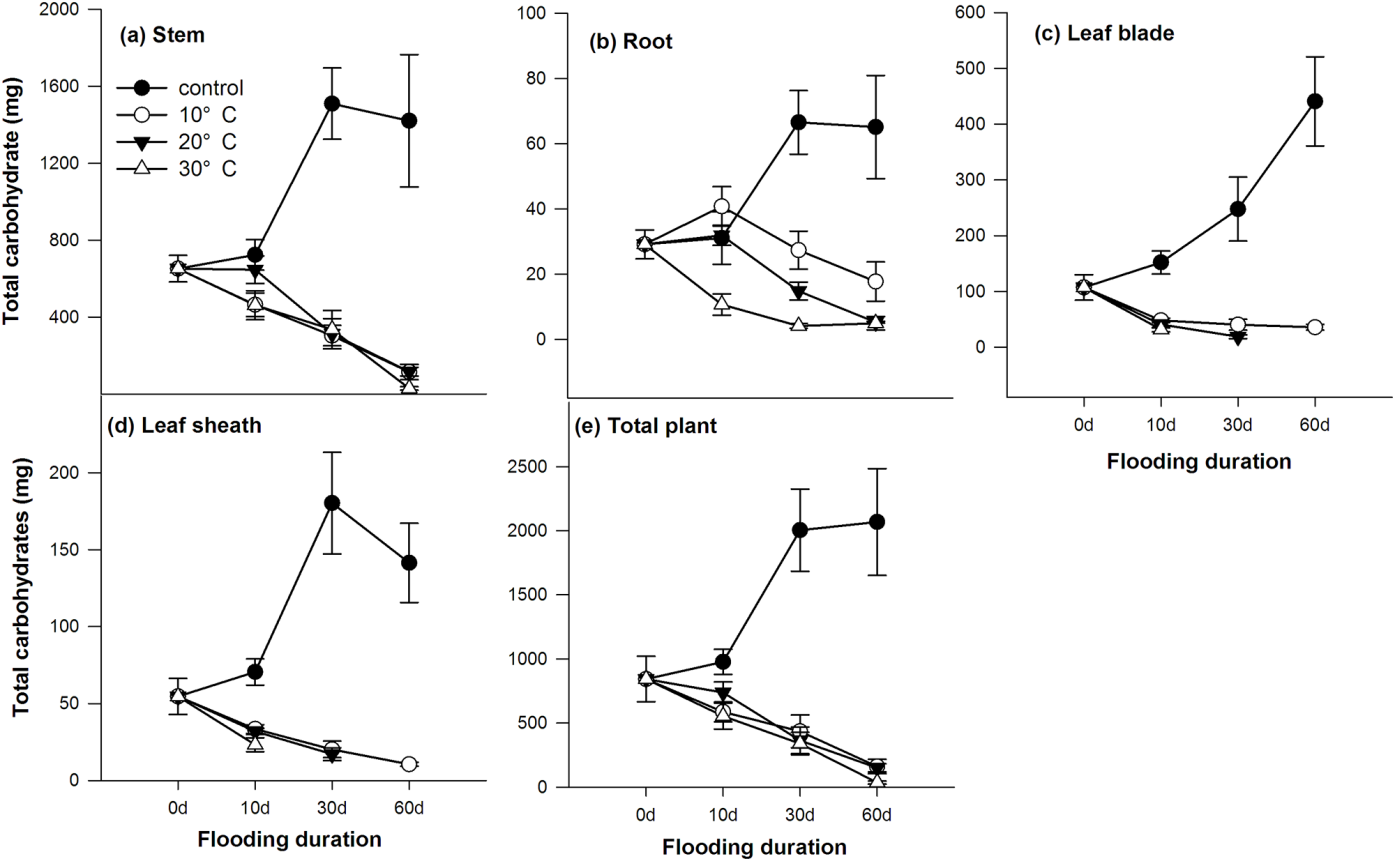
Carbohydrate pools (i.e., the total amount of glucose, fructose, sucrose and starch, g) in the stems (a), roots (b), leaf blades (c), leaf sheathes (d) and whole plants (e, excluding the dead shoot) of *A*. *anomala* either growing in either well-drained soil or submerged at water temperatures of 10°C, 20°C or 30°C in Experiment II. (*n* = 5, points = means ± SE).

Submergence also strongly decreased the total amount of carbohydrates in the individual plants (calculated as the product of the total carbohydrate concentration and total biomass of each organ, including the stem, leaf blade, leaf sheath and root), and this effect was independent of the water temperature (Fig 3). Different water temperatures did not alter the carbon pool size in the stems, leaves and whole plants (p > 0.05, Fig 3a, 3c, 3d and 3e), but a clear difference was found in the roots (p < 0.05, Fig 3b), with a larger pool size at a lower temperature.

**Fig 4:**
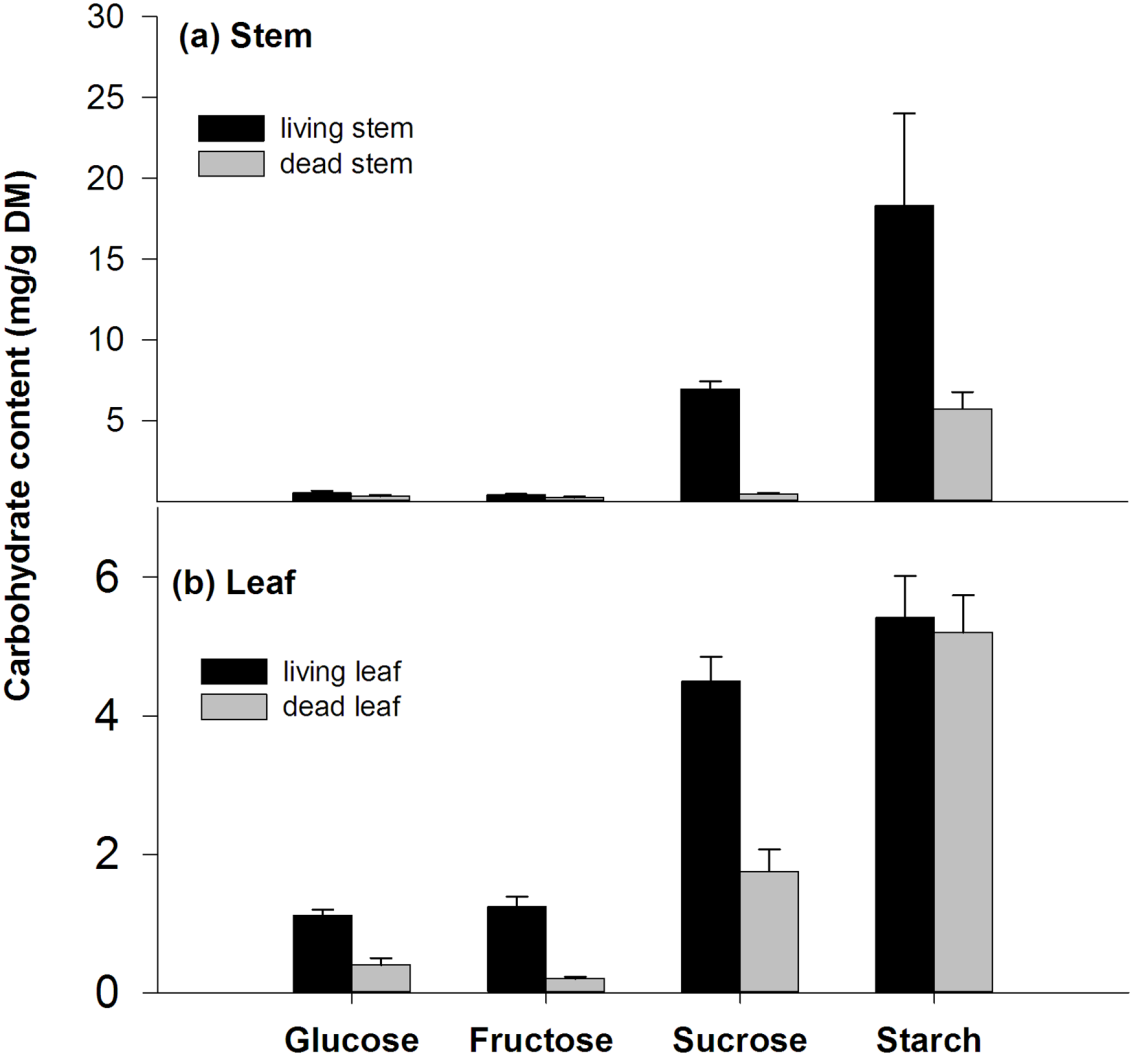
Carbohydrate content (mg·g^-1^ DM) in living stems and dead stems (from plants harvested at day 30 submerged at 30°C) and in living leaves and dead leaves (from plants harvested at day 30 submerged at 30°C). Points = mean + SE, *n = 5*. *, p < 0.05, ns, p > 0.05.

The sucrose and starch content, but not the glucose and fructose content, were significantly higher in the living stem tissues than in the dead stem tissues (Fig 4a). In the leaves, all sugars, but not the starch content, were higher in the living leaf tissues compared to the dead leaf tissues (Fig 4b).

## Discussion

The results have shown that *A*. *anomala* have the abilities to cope with long-term submergence. The studied plants had full survival at water temperature of 10°C and 40% percent survival at 20°C. The results also showed that the submergence tolerance was associated with carbohydrate status and utilization under submergence conditions.

### Submergence caused biomass loss and greatly reduced carbohydrate content

Submergence caused both biomass and carbohydrate loss in submerged *A*. *anomala* plants (Figs 1 and 2). Except for the direct decay of shoots in submergence conditions, carbohydrates loss by respiration might also contribute to the biomass loss, which is an important factor in flood tolerance [16]. As previously reported, the underwater photosynthesis rate of terrestrial plants is extremely low [5], and the contribution to carbohydrate accumulation from underwater photosynthesis could be little, so any dependence of underwater photosynthesis on temperature would not cause the observed effect of submergence on biomass and carbohydrates (Figs 1–4). Thus, we suggested that carbohydrate content in submerged *A*. *anomala* plants were decreased by catabolism activities. This outcome suggested that even when completely submerged, the carbohydrate catabolism system was well-maintained, and carbohydrate utilization permitted survival of long-term submergence at water temperature of 10°C and 20°C. In submerged *A*. *anomala* plants, amylase activity in the stems was similar to or even higher than that of the well-drained plants up to 60 days of submergence [17]. Similarly, starch mobilization has been observed in some submergence tolerant species or tissues, such as the stems of rice [18] and the taproots of *Rumex palustris* [19], but the intolerant species *Daucus carota* could not mobilize starch [12]. Under anoxia, which resembles submergence, amylase activity was induced in anoxia-tolerant tissues such as rice grains and the rhizomes of *Acorus calamus*, but not in intolerant cereal grains and potato tubers [20,21].

On the other hand, despite the substantial reduction of carbohydrates, the decrease was smaller in *A*. *anomala* compared to some species with a different tolerance for submergence or anoxia (S1 Table). The net reduction of soluble sugars was as low as 0.12–0.71 mg·g^-1^ dry mass (DM) per day, whereas for starch it was 0.7–2.4 mg/g DM per day (Fig 2), depending on the water temperature. This conservation of carbohydrates could be due to inhibited growth or restricted metabolism. We did not observe any remarkable growth in submerged *A*. *anomala* plants. No new leaves were produced in contrast to some other tolerant riparian species that still develop new aquatic leaves in water [22], and with this quiescent strategy [23], *A*. *anomala* can tolerate prolonged submergence, which is a natural condition for the species. A study on four other riparian species from the same area showed that submergence tolerance is negatively associated with growth capacity under water [24]. Enhanced survival was also found in rice varieties that have less shoot elongation and less carbohydrate consumption during submergence [25]. In some other species, carbohydrate status has also been referred as a key indicator of flood-tolerance [26,27]. Thus, the slow utilization of carbohydrates, which occurs due to a down-regulated metabolism, might contribute to the high flood-tolerance of *A*. *anomala*.

### Both survival percentage and sugar content in the roots of submerged plants increased with decreasing water temperature

The plant survival percentage was strongly affected by temperature in our submergence experiment, and the correlated changes in both leaf maintenance and root carbohydrates may be a causal association. The survival percentage decreased with duration of submergence, and increased with decreasing water temperature. One of the first causes of death might be the loss of leaf function. Before dying, the submerged *A*. *anomala* plants lost their leaves, and higher temperatures accelerated leaf chlorosis (Fig 1). Leaves continue to play a crucial role for gas exchange when plants are submerged [5]. When water temperature rises, leaves decay more quickly, and the exchange of CO_2_ and O_2_ is even more difficult.

The second cause of death following oxygen deficiency could be the depletion of sugars. To survive during submergence, energy flux from respiration must be maintained for maintenance processes, but high water temperatures would accelerate respiration and carbohydrate utilization [12,28]. We found a heterogeneous distribution of sugars and starch in leaves, stems and roots (Fig 2). Most starch was stored in the stems and leaves of *A*. *anomala* plants and roots had lowest carbohydrate content levels and pool size (Figs 2 and 3). However, a clear difference of carbohydrate pool size was found only in roots from different water temperature treatments (p < 0.05). In contrast, the difference in sugar and starch pool size in stems and leaves would not explain the varying survival percentage according to water temperature (Fig 3). These results suggest that survival in long-term submergence may be more limited by carbohydrates in the roots rather than that in stems and leaves. It was likely that sugar transport from the stems to the roots was probably blocked due to oxygen deficiency in the submerged plants just as in anoxia conditions [29]. These results are also consistent with the findings that combination of high temperature and low soil aeration had more severe effects on root viability of creeping bentgrass than either high temperature or low soil aeration alone [30], probably due to depletion of sugars. Furthermore, the clearer differences of increased glucose, fructose, sucrose content rather than starch with decreased water temperature (p < 0.05, Fig 2) suggested that increased survival rate may be related to higher sugar content, instead of starch content. It should be noted that because the flooding water was frequently changed or air bubbled in our experiments, the submergence conditions in these two experiments resembled river flooding conditions, which is not a fully anoxic environment. Therefore, the flooded plants might suffer less post-anoxic stress than plants in fully anoxic conditions.

## Conclusions

*A*.*anomala* is characterized by high flooding tolerance and sustained capability to mobilize carbohydrate pool exposed to submergence. The survival percentage and carbohydrate status of submerged *A*. *anomala* plants were remarkably improved by a lower water temperature. Increased sugar content rather than starch content seemed to contribute to higher submergence tolerance. The carbohydrate pool size of root seemed to be critical for survival of submergence than that of stems and leaves for *A*.*anomala* plants. These results suggested that survival of submerged plants can be greatly enhanced during the reservoir’s prolonged winter flooding when temperatures are lower, which may be beneficial for vegetation development and protection in the riparian area of the three Gorges reservoir.

## Supporting information

S1 Data Data for the paper. (XLS)

S1 Fig Average daily temperature (°C) (dotted line) in each month in the TGR region and water table (m) before (open square, realistic river water surface altitude is less than 100 m) and after (closed square) Three Gorges Reservoir (TGR) construction. (TIF)

S1 Table Carbon utilization rates of different species exposed to anoxia or submergence conditions (percent/day, the percent reduction of carbohydrate content in mg·g^-1^ biomass from the initial value at day 0 and averaged by treatment duration). (DOC)
