# Supplementary figures and images for: Improved flooding tolerance and carbohydrate status of flood-tolerant plant Arundinella anomala at lower water temperature
Source: PLoS One. 2018 Mar 21;13(3):e0192608. doi: 10.1371/journal.pone.0192608 (PMC5862403; doi:10.1371/journal.pone.0192608)

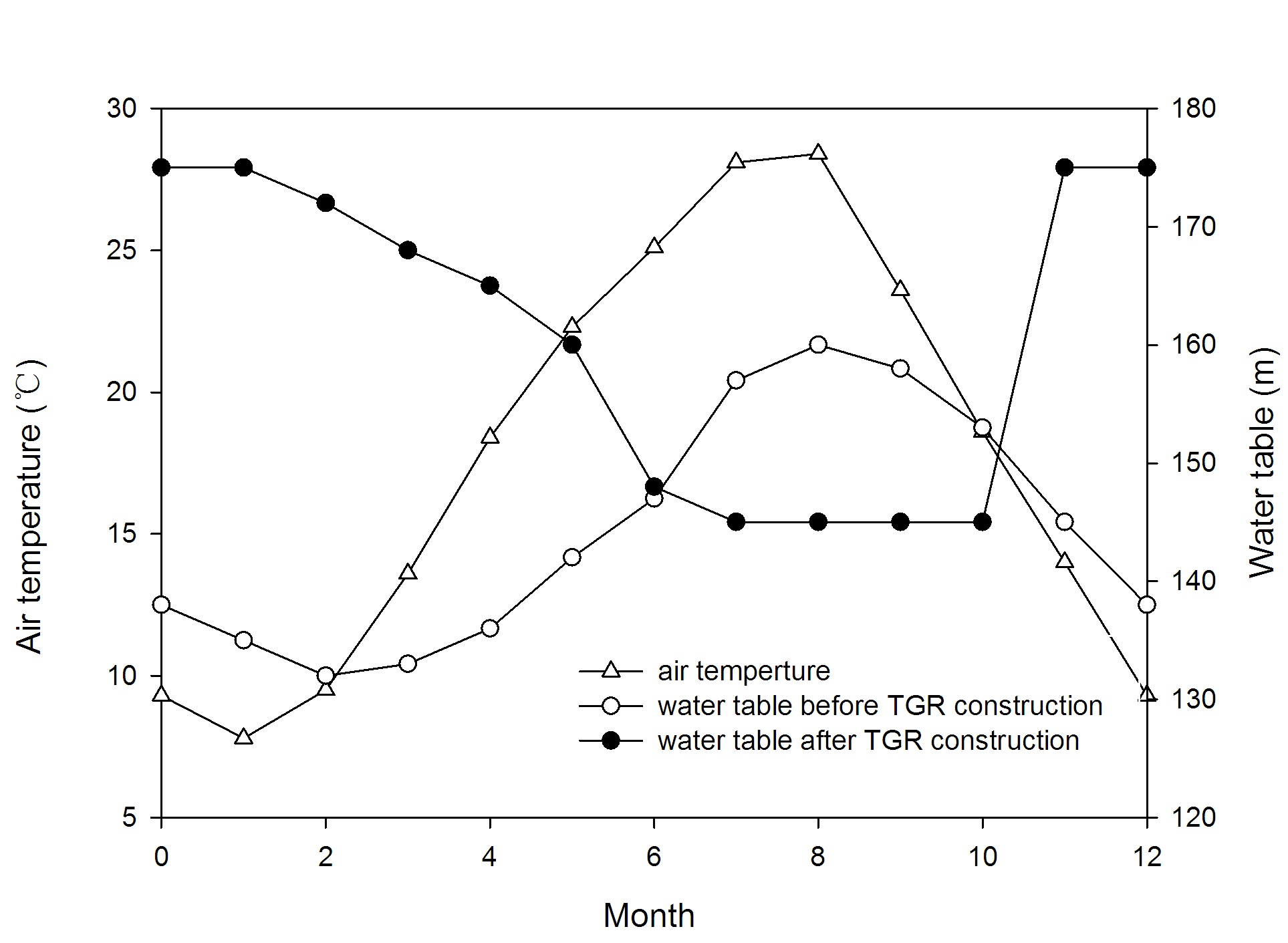

Supplement: S1 Fig — (TIF) [file pone.0192608.s002.TIF]
