# Supplementary material for: Improved flooding tolerance and carbohydrate status of flood-tolerant plant Arundinella anomala at lower water temperature
Source: PLoS One. 2018 Mar 21;13(3):e0192608. doi: 10.1371/journal.pone.0192608 (PMC5862403; doi:10.1371/journal.pone.0192608)
Supplement: S1 Table — (DOC) [file pone.0192608.s003.doc]

**S1 Table**

| **Species** | **Anoxia/ flooding tolerance** | **Utilization rate**  (mg·g^-1^ dry mass) | **Literature** |
| --- | --- | --- | --- |
| *Arundinella anomala* | high | 1.2–1.3 | In this experiment |
| *Acorus calamus* | high | 100 | Schlüter and Crawford 2001 |
| *Scirpus maritumus* | high | 0.5 | Barclay and Crawford 1983 |
| *Phlaris arundinacea* | medium | 0.6 | Barclay and Crawford 1983 |
| *Iris pseudacorus* | medium | 1.4 | Schlüter and Crawford 2001 |
| *Oryza sativa* | low to medium | 2.5–10.1 | Singh, Singh and Ram 2001 |
| *Glyceria maxima* | low | 11.4 | Barclay and Crawford 1983 |
| *Triticum aestivum* | very low | 9.1 | Mustroph and Albrecht 2003 |
| *Zea mays* | very low | 15.6 | Mustroph and Albrecht 2003 |
